# Novel findings on the mitochondria in ciliates, with description of mitochondrial genomes of six representatives

**DOI:** 10.1007/s42995-024-00249-7

**Published:** 2024-09-23

**Authors:** Tengteng Zhang, Jinyu Fu, Chao Li, Ruitao Gong, Khaled A. S. Al-Rasheid, Naomi A. Stover, Chen Shao, Ting Cheng

**Affiliations:** 1 Laboratory of Biodiversity and Evolution of Protozoa in Wetland, College of Life Sciences, Shaanxi Normal University, Xi’an, 710119 China; 2 Key Laboratory of Evolution & Marine Biodiversity (Ministry of Education), and Institute of Evolution & Marine Biodiversity, Ocean University of China, Qingdao, 266003 China; 3 Zoology Department, College of Science, King Saud University, Riyadh, 11451 Saudi Arabia; 4 Department of Biology, Bradley University, Peoria, IL 61625 USA

**Keywords:** Mitogenome, Spirotrichea, Evolution, Split gene, Synteny, Codon bias

## Abstract

Determining and comparing mitochondrial genomes (mitogenomes) are essential for assessing the diversity and evolution of mitochondria. Ciliates are ancient and diverse unicellular eukaryotes, and thus are ideal models for elucidating the early evolution of mitochondria. Here, we report on six new mitogenomes of spirotrichs, a dominant ciliate group, and perform comparative analyses on 12 representative species. We show that: (1) the mitogenomes of spirotrichs are linear structures with high A+T contents (61.12–81.16%), bidirectional transcription, and extensive synteny (except for the *nad5*, *ccmf* and *cob* genes in Euplotia); (2) the non-split of NADH dehydrogenase subunit 2 gene (*nad2*) is a plesiomorphy of ciliates, whereas it has evolved into a split gene in Spirotrichea (apart from *Euplotes* taxa), Oligohymenophorea, and Armophorea; (3) the number of small subunit ribosomal proteins (rps) encoded in mitogenomes increases in the later branching classes of ciliates, whereas *rps8* shows a loss trend during the evolution of *Euplotes* taxa; (4) the mitogenomes of spirotrichs exhibit A/T codon bias at the third position, and the codon bias is mainly due to DNA mutation in oligotrichs, hypotrichs and *Diophrys appendiculata*; (5) the phylogenetic position of *D. appendiculata* is unstable and controversial based on both phylogenetic analyses and mitogenome evidence. In summary, we investigated the mitogenome diversity of spirotrichs and broadened our understanding of the evolution of mitochondria in ciliates.

**Supplementary Information:**

The online version contains supplementary material available at 10.1007/s42995-024-00249-7.

## Introduction

Semiautonomous mitochondria possessing their own genomes (mitogenomes) are essential organelles for nearly all eukaryotes, with diverse functions including energy supply, apoptosis, homeostasis, and metabolism (Chen et al. 2022; Gawryluk et al. 2016; Müller et al. 2012; Stairs et al. 2021). In eukaryogenesis, after acquisition of the endosymbiotic mitochondrion cenancestor (mitochondrion of the last eukaryote common ancestor), mitochondria evolution has played many important roles in the evolution of eukaryotes (Archibald 2014; Muñoz-Gómez et al. 2022; Roger et al. 2017; Sagan 1967). Practically, the high copy number and rapid evolution of mitochondrial genes have led researchers to use them for “barcoding” studies, in which genes such as *cox1* (cytochrome c oxidase subunit 1 gene) and mtSSU rDNA (mitochondrial small subunit ribosomal RNA gene) are used to reflect the evolutionary relationships of eukaryotes (Lian et al. 2023; Zhang et al. 2019). Despite this interest in mitochondrial evolution, much of our knowledge has been limited to plants and animals (Prada et al. 2023; Shatskaya et al. 2023), resulting in insufficient understanding of the diversity and early evolution of mitochondria (e.g., through the study of single-celled eukaryotes).

Ciliates are good model single-celled eukaryotes to extend our work on mitogenomes, as they are a monophyletic phylum group, inexpensive to store and culture, have rapid generation times, are easy to manipulate in experiments, and maintain stable characteristics over many generations (e.g., Montagnes et al. 2012). These features have made ciliates valuable experimental organisms in many areas of research (Lu et al. 2023; Rotterová et al. 2020; Tang et al. 2023; Tian et al. 2022; Wei et al. 2022; Zhang et al. 2023; Zheng et al. 2024; Zhou et al. 2024). Furthermore, as an ancient eukaryotic lineage, ciliates have been used to explore the early evolution of mitochondria and mitogenomes (Huang et al. 2021b; Johri et al. 2019; Watt et al. 2019). Among the ciliates, the class Spirotrichea has the richest available mitogenome data (14 species, covering ten genera and three subclasses), making them ideal subjects for mitogenomic comparative analyses and mitochondrion evolutionary studies (Huang et al. 2021a; Zhang et al. 2021).

Here, by applying high-throughput sequencing technology to assess the comparative analyses of mitogenomes, we report on six new mitogenomes of spirotrichs (from *Pseudokeronopsis carnea*, *Pseudokeronopsis flava*, *Euplotes vannus*, *Euplotes crassus*, *Euplotes raikovi*, and *Diophrys appendiculata*). In doing so, we elucidate their structure, codon usage, and diversity. The mitogenomes, along with published mitogenomes from representative spirotrichs, were then used to perform a class-wide comparative analysis. Finally, phylogenomic work based on mitogenomes was used to clarify the evolutionary relationships of ciliates.

## Materials and methods

### Ciliate collection, identification, and culturing

*Pseudokeronopsis carnea* and *P. flava* were collected from a freshwater pond in Baihuayuan Park (36°04′ N, 120°22′ E), Qingdao, China. *Euplotes vannus* and *E. raikovi* were collected at Silver Beach (35°55′ N, 120°12′ E), Qingdao, China. *Euplotes crassus* was supplied by Prof. Adriana Vallesi of University of Camerino. *Diophrys appendiculata* was collected from Zhanqiao Beach (36°05′ N, 120°32′ E), Qingdao, China. All six species were identified based on morphological features, observed in vivo and from protargol stains (Pan et al. 2013). For each sample, several cells were isolated from the original culture and washed five times in sterile water. The cells were then transferred to Petri dishes and cultured in about 30 ml filtered and autoclaved freshwater (two *Pseudokeronopsis* taxa) or marine water (remaining four species) at 25 ℃, with food of *Escherichia coli* or a natural assemblage of microbes introduced with rice grains.

### Genomic DNA extraction and Illumina sequencing

After two days of starvation by treating with the antibiotic–antimycotic (2 mL/L, ThermoFisher Scientific, USA; Cat No. 15240062), cultures were harvested by picking cells manually (*P. carnea* and *P. flava*) or filtering with a 5 µm membrane (*E. vannus*, *E. crassus*, *E. raikovi*, and *D. appendiculata*) (Shanghai Xin Ya Purification Equipment, Shanghai, China). Genomic DNA was extracted using the MasterPure™ Complete DNA & RNA Purification Kit (Lucigen, USA; Cat No. 15517) for the two *Pseudokeronopsis* species and the DNeasy Blood & Tissue Kit (Qiagen, Hilden, Germany; Cat No. 69506) for *E. vannus*, according to manufacturers’ instructions. Genomic DNA extraction for the remaining three samples was performed using a standard Phenol–Chloroform protocol (Di Pietro et al. 2011). All DNA samples were treated with RNase A (Omega Bio-tek, Norcross, GA, USA; Lot No. L10RG) to eliminate RNA contamination.

For each genomic DNA sample, sequencing libraries of 350 bp were constructed using the NEBNext® Ultra™ DNA Library Prep Kit (NEB, Ipswich, MA; Cat No. E7370L) (Jin et al. 2023; Lyu et al. 2023; Zheng et al. 2021). These libraries were paired-end sequenced for 150 bp on an Illumina HiSeq4000 sequencer (Novogene, Beijing, China). Each sequencing dataset was assembled using SPAdes v.3.11 with −*k* = 21, 33, 55, 77, after removal of adapters and low-quality reads, according to Li et al. (2021) and Lyu et al. (2024).

### Mitogenome assembly and annotation

The six new genome assemblies were each searched with blastn using reference genomes as query (*e*-value = <1e−5) according to Zhang et al. (2021). A single linear sequence of mitogenome was discovered in *E. vannus* and *D. appendiculata*, whereas multiple contigs were found for mitogenomes of the other isolates (*P. carnea*: five contigs, *P. flava*: two contigs, *E. crassus*: 12, *E. raikovi*: 11 contigs). For *P. flava*, two mitochondrial contigs were manually assembled into one linear structure. For *P. carnea*, *E. crassus*, and *E. raikovi*, the mitochondrial contigs were first assembled by SeqMan v.7.1.0 (DNAStar). Polymerase chain reactions (PCRs) were then carried out with 2 × EasyTaq® PCR SuperMix (Transgen Biotech, China; Cat No. AS111-14) to close the remaining gaps and to support the accuracy of these mitogenome sequences. Primers were designed based on the obtained mitochondrial contigs (Supplementary Table S1). The PCR conditions, PCR product purification, and cloning were all conducted according to methods described by Zhang et al. (2021). The cloned PCR fragments were sequenced at Tsingke Biological Technology Company (Beijing, China) and assembled by SeqMan v.7.1.0 (DNAStar). Finally, the raw read data were used to remap onto the mitogenomes, obtaining average sequencing depths of the mitogenomes as follows: 1609 for *P. carnea*; 2794 for *P. flava*; 2159 for *E. vannus*; 10,299 for *E. crassus*; 4062 for *E. raikovi*; 1191 for *D. appendiculata* (Supplementary Fig. S1).

Each newly sequenced mitogenome was annotated by combining the results of MITOS, MFannot, and NCBI Open Reading Frame Finder (https://www.ncbi.nlm.nih.gov/orffinder) with genetic code 4 according to Zhang et al. (2021). Subsequently, protein-coding genes and ribosomal RNA (rRNA) genes were identified by blastp and blastn searches, respectively, and were compared against the NCBI non-redundant database. tRNAscan-SE v.2.0 was used for transfer RNA (tRNA) gene prediction (Chan and Lowe 2019). Six mitogenome maps were constructed using CGView Server (Grant and Stothard 2008).

To illustrate the structures of NADH dehydrogenase subunit 2 (nad2) proteins within the Spirotrichea, the transmembrane helix structures were first predicted by TMHMM v.2.0 (Krogh et al. 2001). Second, the nad2 protein sequences were aligned by Bioedit v.7.2.5 (Hall 1999). Third, the nad2 protein sequences were analyzed by HHpred, which is used for the detection of distant protein homology and 3D structure prediction through hidden Markov model (Zimmermann et al. 2018).

### Phylogenetic analyses

All mitochondrial protein sequences with known functions were combined with the orthologous proteins of the relative ciliates, forming 29 datasets with two species of apicomplexan as outgroup. For *E. vannus* and *E. crassus*, two populations of each species were included in this study. The newly sequenced populations that we isolated were named “new” (*Euplotes vannus*_new and *Euplotes crassus*_new). The previously reported ones were named “old” (*Euplotes vannus*_old with accession number MT665959 and *Euplotes crassus*_old with accession number GQ903131). Each of the 29 datasets was aligned on the GUIDANCE2 Server with the default parameters (Sela et al. 2015), and then trimmed by trimAl v.1.4 (Capella-Gutiérrez et al. 2009). Finally, all of the alignments were concatenated with PhyloSuite v.1.2.3 (Zhang et al. 2020), generating a final dataset for phylogenetic analyses. The small subunit ribosomal RNA gene (SSU rDNA) sequences of corresponding species mentioned above were downloaded from the GenBank database (Supplementary Table S2). The SSU rDNA dataset was aligned by MAFFT v.7 (Katoh et al. 2019) and trimmed using Bioedit v.7.2.5 (Hall 1999).

The Maximum Likelihood (ML) tree of mitochondrial data was constructed with 1000 replicates by IQ TREE v.1.5.5 (Nguyen et al. 2015) using the model of LG+C60+F+G4. The ML tree of SSU rDNA was built with 1000 replicates by RAxML-HPC2 on ACCESS v.8.2.12 (Stamatakis 2014) in CIPRES Science Gateway. The Bayesian interference (BI) analyses of the two datasets were carried out with 10,000,000 generations using MrBayes on XSEDE v.3.2.7 (Ronquist et al. 2012) in CIPRES Science Gateway under the LG+I+G+F (mitochondrial data) and GTR+I+G (SSU rDNA) model, respectively. For both datasets, the sampling frequency was 100 generations with a burn-in of 25,000 trees. FigTree v.1.4.4 was used for the visualization of tree topologies (http://tree.bio.ed.ac.uk/software/figtree/).

To further assess the phylogenetic position of *D. appendiculata*, a constrained ML tree was built by forcing the cluster of *D. appendiculata* and *Euplotes* spp. Then to test the prediction that *D. appendiculata* clusters with *Euplotes* spp., the approximately unbiased (AU) test (*α* = 0.05) was performed according to Zhang et al. (2022).

### Codon usage analyses

All protein-coding genes, including known proteins and unknown ORFs (open reading frames), were selected for codon usage analyses in the 12 Spirotrichea species. The RSCU (relative synonymous codon usage), GC12 (the mean G+C content at the first and second positions of all codons), GC3 (the G+C content at the third position of all codons), GC3s (the G+C content at the third position of synonymous codons), and actual ENC (effective number of codons) values were calculated using CodonW v.1.4.2 (ftp://molbiol.ox.ac.uk/cu/codonW.tar.Z). A heatmap of RSCU was generated by TBtools (Chen et al. 2020). The neutrality plot (i.e., GC12-GC3) and regression line were drawn using the ggplot2 R-package (http://github.com/hadley/ggplot2). The regression analysis and *t* test (to test if the slope significantly differed from zero, *α* = 0.05) were performed by RStudio v.1.3.1093 (https://docs.posit.co/previous-versions/rstudio.html) and Graphic Pad Prism v.8.0 (https://www.graphpad.com), respectively. The ENC plot was drawn by the ggplot2 R-package. The expected ENC values and standard curve were calculated according to Huang et al. (2021a).

## Results

### Structures and features of newly sequenced mitogenomes

**Fig. 1:**
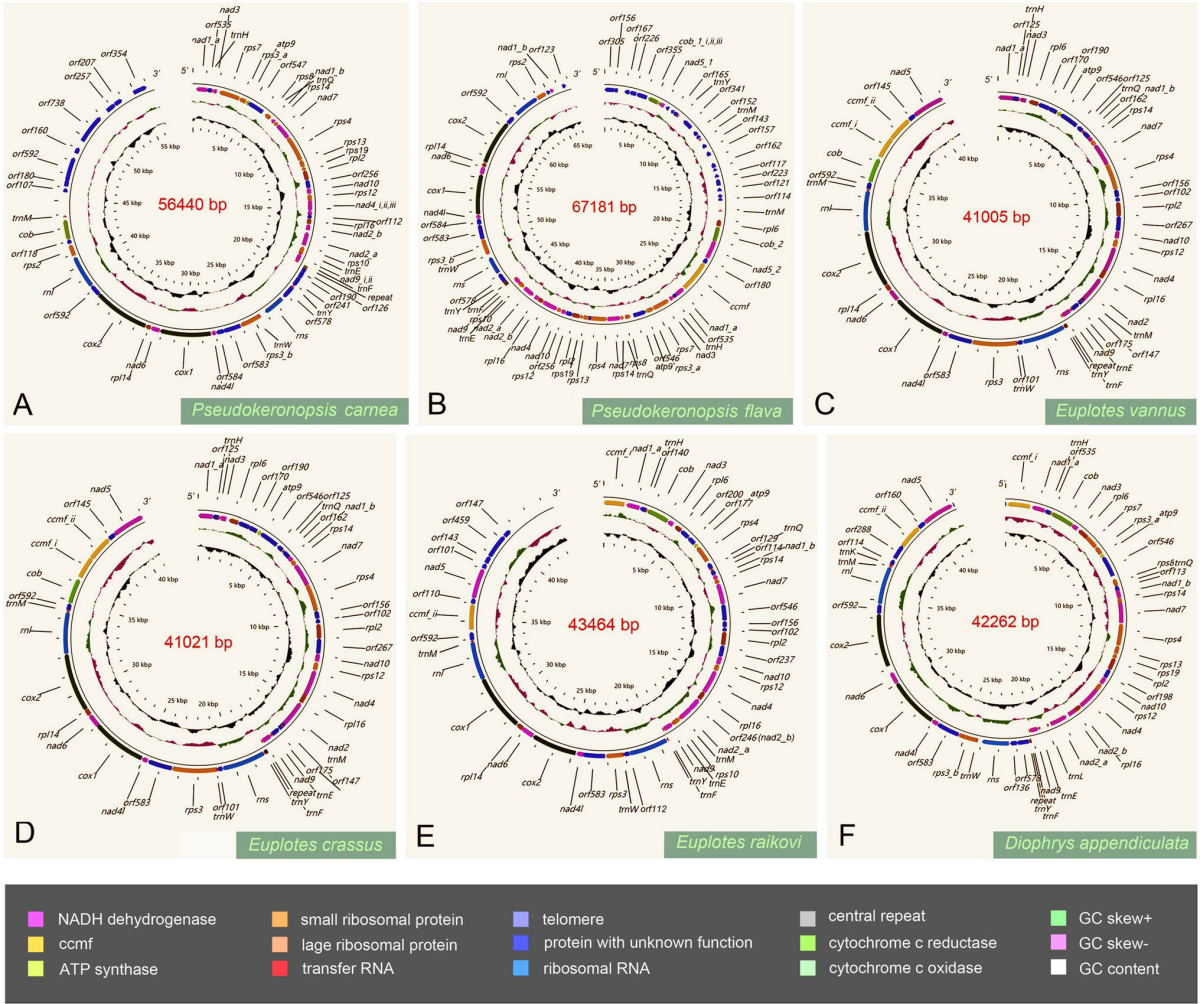
Newly characterized mitogenome structures of six spirotrich species. Genes are represented by different colored blocks with arrows indicating transcription directions. Outside and inside blocks of each mitogenome map indicate the genes on the forward and reverse strands, respectively

All six newly sequenced mitogenomes were linear structures with genome sizes ranging in size from ~41,000 to 67,000 bp (Fig. 1). Among them, only that of *D. appendiculata* was completely sequenced, based on the detection of telomere repeats at both ends. The length and A+T content of the mitogenomes of the two *Pseudokeronopsis* isolates were large compared to those of four euplotids (Table 1). All six mitogenomes use two transcriptional directions and have an AT-rich central repeat region as follows: 158 bp for *E. vannus*, 172 bp for *E. crassus*, 22 bp for *E. raikovi*, and 92 bp for *D. appendiculata* (Fig. 1; Table 1). For *P. carnea*, considering that an estimated gap region (95 bp) by SPAdes is detected in the repeat sequence, the length of this region could not be determined but is estimated to be 157 bp. Similarly, the gap between two mitogenome contigs of *P. flava* is located in the central repeat region, and we connected them manually. Therefore, the central repeat length of *P. flava* should be equal or greater than 333 bp. For each species, the central region is composed of repeated sequence units, and the number of repeat units in two species of *Pseudokeronopsis* is unknown (Table 1). Mitochondrial genes are transcribed from the central repeat region (between the *trnF* and *trnY* genes) toward the two ends for all six taxa (Fig. 1). No terminal inverted repeats or introns are found in these mitogenomes (Table 1).

**Table 1:** Mitogenome information within the class Spirotrichea

Species	Accession No.	Genome size (bp)	A+T content (%)	Telomere	Central repeat	Terminal inverted repeat (bp)
*Strombidium* cf. *sulcatum*	MT471316	54,912	71.68	TTATATCCTTTCTCCCCTATATCTCTATAGTACT (both ends)	ATAAATTTAATTTTA (2)+ irregular sequence for the rest	*
*Oxytricha trifallax*	JN383843	69,800	76.17	CGACTCCTCTATCCTCATCCTAGACTCCGCTTACT (both ends)	TATATAAA (11)+ TATAAATAAA (3)+ AAAAAG (5)	~1800
*Laurentiella strenua*	KX529838	66,721	75.75	CCTACTACGCTTCATACGCTAAA (partial) (both ends)	ATATAAATGTATATAA (7)+ ATAAA(TA)*n*T (49)+ TTT(AT)*n* (4), *n* = 0–8	~2400
*Urostyla grandis*	KX494929	60,924	61.12	GTAGCACATGTAG (3′ end)	ATATATTTATTAATATATAGTA (10)	*
*Pseudokeronopsis carnea*	**PP853579**	56,440	79.93	*	TAATAAA(TA)4 (*n*)	*
*Pseudokeronopsis flava*	**PP853578**	67,181	81.16	*	[TAATAAA(TA)5, 6] (*n*)	*
*Euplotes vanleeuwenhoeki*	MK889230	41,682	74.91	*	CATCATTTGTRGTGTATA (16)	*
*Euplotes minuta*	GQ903130	41,978	64.74	*	ATAGTATATAATGTATAC (63)+ ATAGTATATAATGTTAC (1)+ ATAGTATATAATTGTTAC (18)	*
*Euplotes raikovi*	**PP853577**	43,464	71.43	*	ATAGTATATAATGTATAC (1)	*
*Euplotes vannus*	**PP853575**	41,005	66.09	*	TATACATTATATACTATG (8)	*
*Euplotes crassus*	**PP853576**	41,021	66.15	*	TATACATTATATACTATG (9)	*
*Diophrys appendiculata*	**PP808730**	42,262	73.12	CTATACTCCGCTTAGCTACTTGCCGCTACG (both ends)	TATATTTATATATATA (4)	*

The newly characterized mitogenomes are in bold

The ^*^ denotes absence. The numbers in brackets indicate the repeat numbers of the repeated unit

The gene components of the six mitogenomes are almost identical to those of previously reported ciliates, including ribosomal RNA (rRNA: *rns*, small subunit ribosomal RNA gene; *rnl*, large subunit ribosomal RNA gene), transfer RNA (tRNA), protein-coding genes with known functions, and ORFs with unknown functions (Zhang et al. 2021). Among these, the protein-coding genes with known functions are mainly composed of NADH dehydrogenase gene (*nad*), cytochrome c oxidoreductase gene (*cob*, *cox1–2*), cytochrome c maturation related gene (*ccmf*), ATP synthase gene (*atp9*), and small/large subunit ribosomal protein genes (*rps*, *rpl*) (Figs. 1, 3). In the mitogenome of *P. carnea*, 29 known protein-coding genes were identified. *P. flava* has 26 known protein-coding genes and *D. appendiculata* has 27. For the other three *Euplotes*, *E. vannus* and *E. crassus* have 26 protein-coding genes, while *E. raikovi* has 27 (Fig. 1). The mitochondrial tRNA gene components are similar across all species (Fig. 1; Table 2).

**Table 2:** The tRNA genes of mitogenomes in Spirotrichea

Species	*trnGln* (*Q*)	*trnLeu* (*L*)	*trnGlu* (*E*)	*trnPhe* (*F*)	*trnTyr* (*Y*)	*trnTrp* (*W*)	*trnLys* (*K*)	*trnMet* (*M*)	*trnHis* (*H*)	*trnCys* (*C*)
*Strombidium* cf. *sulcatum*	+	+	+	+	+	+	+	+^a^	*	*
*Oxytricha trifallax*	+	+	+	+	+	+	+	+^a^	+	+^a,b^
*Laurentiella strenua*	+	+	+	+	+	+	+	*	*	*
*Urostyla grandis*	+	*	+	+	+	+	*	*	+^a^	*
*Pseudokeronopsis carnea*	+	*	+	+	+	+	*	+	+	*
*Pseudokeronopsis flava*	+	*	+	+	+^a^	+	*	+^a^	*	*
*Euplotes vanleeuwenhoeki*	+	*	+	+	+	+	*	+	+	*
*Euplotes minuta*	+	*	+	+	+	+	*	+	+	*
*Euplotes vannus*	+	*	+	+	+	+	*	+^a^	+	*
*Euplotes crassus*	+	*	+	+	+	+	*	+^a^	+	*
*Euplotes raikovi*	+	*	+	+	+	+	*	+^a^	+	*
*Diophrys appendiculata*	+	+	+	+	+	+	*	+	+	*

Capital letters in brackets are abbreviations of the tRNA genes. The “*” denotes absence and “+” denotes presence. The “a” represents that there are two copies for the target gene. The “b” represents that one of the two copies of *trnC* is a pseudogene

### Mitochondrial gene synteny and rearrangement within Spirotrichea

**Fig. 2:**
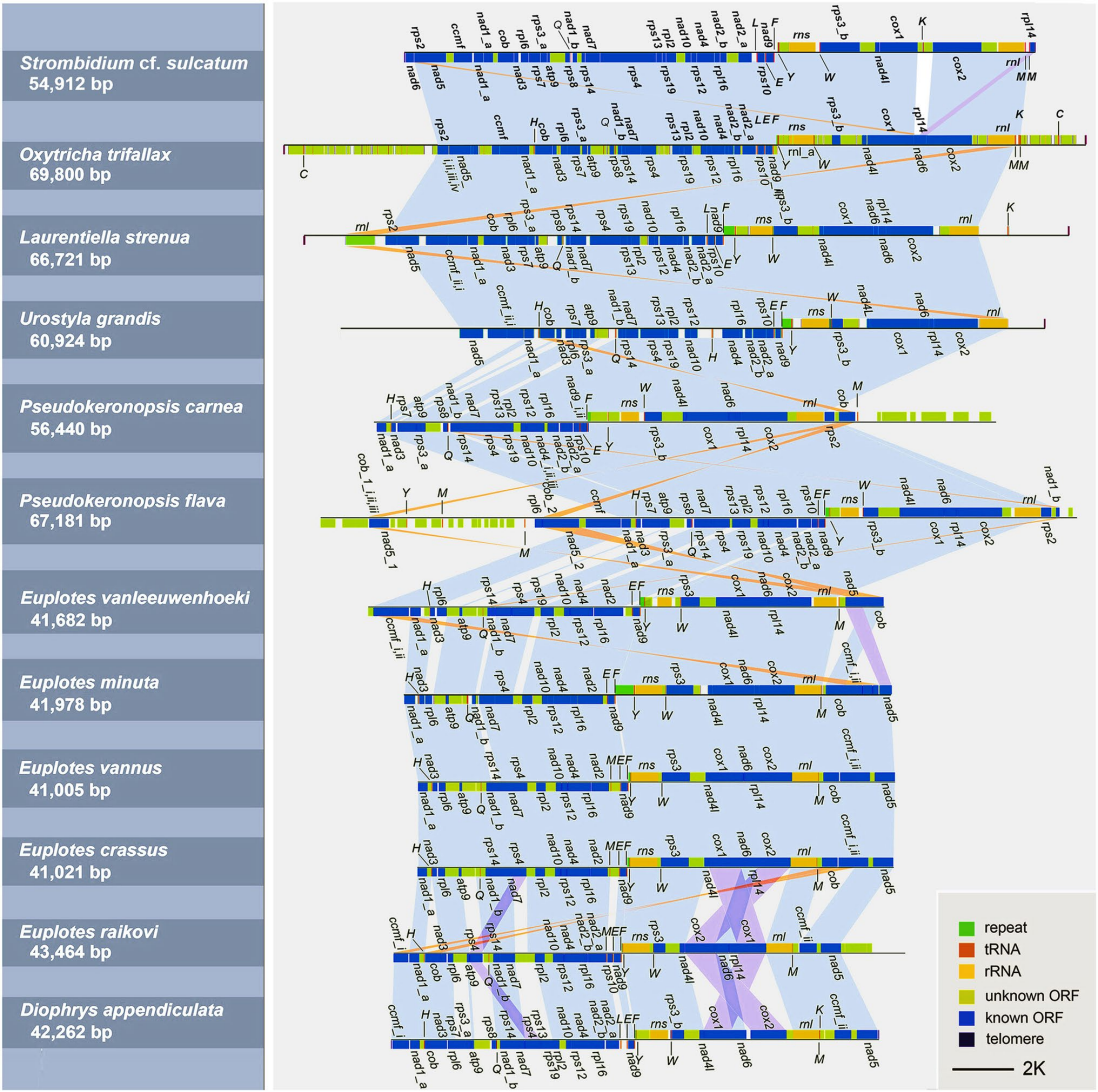
Mitogenome synteny of representative species within the class Spirotrichea. The blue, purple, and pink shadows indicate collinearity, translocation, and inversion of genes, respectively

Comparative analyses performed between the six newly characterized mitogenomes with those of representative spirotrichs (*Strombidium* cf. *sulcatum*, *Oxytricha trifallax*, *Laurentiella strenua*, *Urostyla grandis*, *Euplotes vanleeuwenhoeki*, *Euplotes minuta*) show that all 12 mitogenomes of Spirotrichea are linear, with a high A+T content (Table 1). Among the 12 species studied here, only four mitogenomes (*S.* cf. *sulcatum*, *O. trifallax*, *L. strenua*, and *D. appendiculata*) are completely sequenced (capped with two telomeres). In addition, the Hypotrichia ciliates appear to possess larger mitogenomes than Oligotrichia and Euplotia taxa (Table 1). The bidirectional transcription of mitochondrial genes from a central repeat region is a common feature in spirotrichs (Fig. 2). However, the central repeat units are divergent among genera and even species. For example, two *Pseudokeronopsis* species have similar repeat units (Table 1). Among the five *Euplotes* species, three kinds of repeat structures are observed: (1) the same repeat unit is shared between *E. vannus* and *E. crassus*; (2) *E. minuta* and *E. raikovi* also share a common repeat unit; (3) *E. vanleeuwenhoeki* has a unique repeat sequence (Table 1). Finally, the former two types of unit sequences (i.e., unit type of *E. vannus* + *E. crassus*, and unit type of *E. minuta* + *E. raikovi*) are reverse complementary to each other (Table 1).

**Fig. 3:**
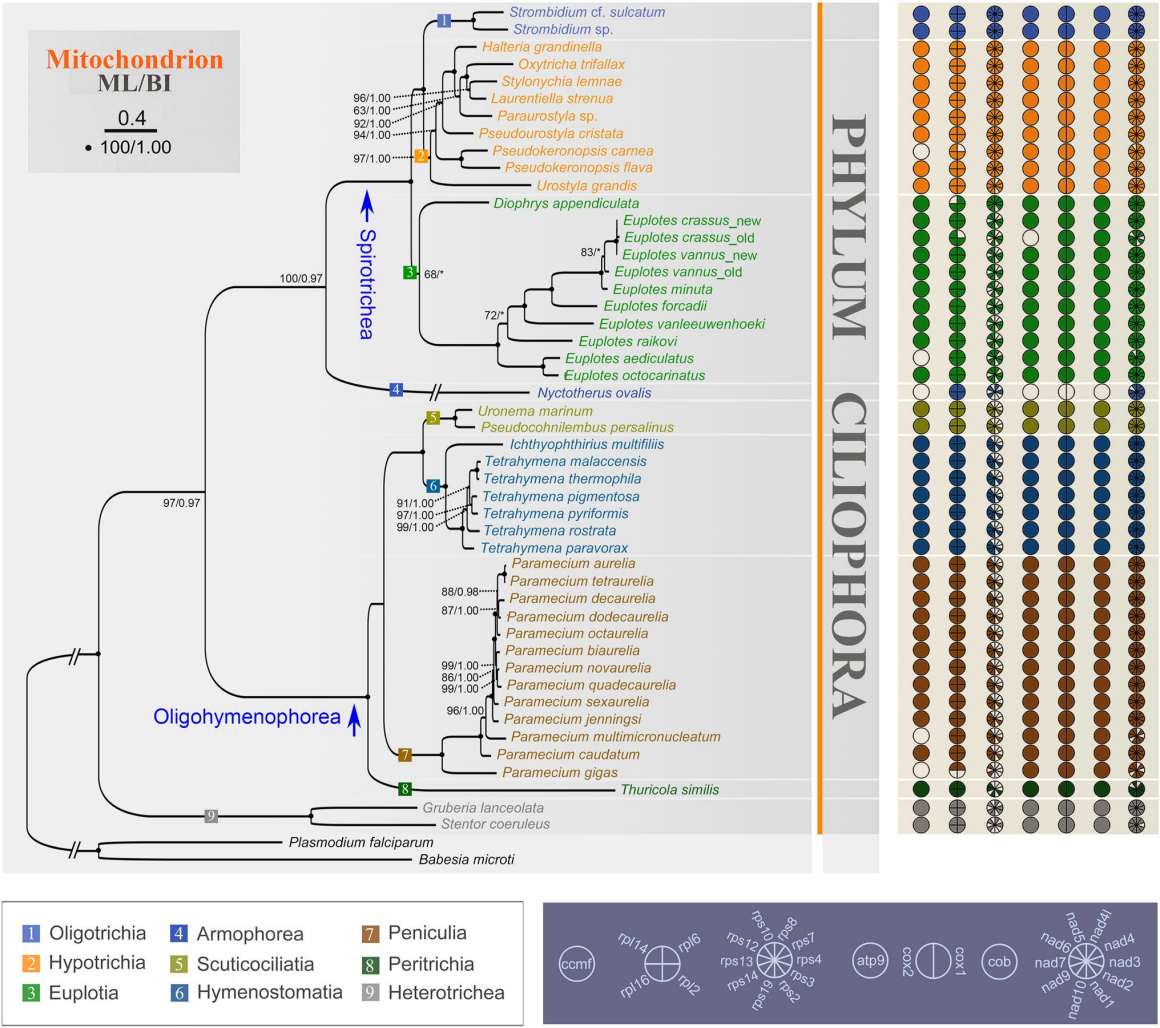
Maximum Likelihood (ML) tree focusing on the aerobic ciliates based on the concatenated mitochondrial data and the components of mitochondrial protein-coding genes. Numbers at the nodes represent the bootstrap values of ML and the posterior probability values of Bayesian analysis (BI). Asterisks (*) indicate disagreement between the ML and BI methods. The scale bar corresponds to 40 substitutions per 100 nucleotide positions. Regarding the pie charts to the right of the tree diagram, solid circles represent the presence of protein-coding genes, while blank circles indicate the absence of genes

In general, the set of protein-coding genes with known functions is identical in these taxa, with the following main structural differences (Figs. 2, 3): (1) the *nad2* gene is split into two parts (*nad2_a*, *nad2_b*) in *S.* cf. *sulcatum*, *D. appendiculata*, *E. raikovi*, and the five hypotrichs, while it is not split in the remaining *Euplotes* species; (2) *P. carnea* lacks the *nad5* and *ccmf* genes in comparison with other ciliates, possibly due to incomplete sequencing of this mitogenome; (3) *rps2* gene is absent in both species in the Euplotia and in *U. grandis* (both of which are also incompletely sequenced); (4) the *rps7* gene is absent in all *Euplotes* isolates, and *rps10* gene is only absent in *E. vanleeuwenhoeki*; (5) *rps8* gene is missing in *E. vannus*, *E. crassus*, and *E. minuta*; (6) *rpl6* gene is missing in *P. carnea* and *E. crassus*_old, while *rpl14* gene is only absent in *D. appendiculata*.

The *trnQ*, *trnE*, *trnF*, *trnY*, and *trnW* genes are conserved in all of the species (Table 2). In addition, *trnL* is detected in the four completely sequenced mitogenomes, while *trnK* is only present in *S.* cf. *sulcatum*, *O. trifallax*, and *L. strenua*. This comparison also shows that *trnM* is missing in the mitochondria of *U. grandis* and *L. strenua*, and *trnH* is missing in the mitochondria of *S.* cf. *sulcatum*, *P. flava*, and *L. strenua*. The mitochondrial rRNA genes (*rns* and *rnl*) are more conserved when compared with the tRNA and protein-coding genes of these mitogenomes. Only *L. strenua* shows a unique feature, as it has two copies of *rnl*, which are transcribed in opposite directions (Fig. 2).

Our analyses also reveal that the mitogenomes of these 12 Spirotrichea ciliates exhibit extensive gene collinearity (Fig. 2). The notable differences in gene order between Oligotrichia and the orders Hypotrichia and Euplotia are an inversion of *nad6* gene and a transposition of *rpl14* gene. The gene order in the mitochondrion of *P. carnea* is consistent with other hypotrichs, except for an inversion of the *cob* gene. Both the inversion of *nad1_b* gene and duplication of *cob* and *nad5* genes are detected in *P. flava*. Moreover, in contrast to *U. grandis*, *O. trifallax*, and *L. strenua*, the *cob* and *rpl6* genes of *P. flava* are translocated. Aside from the differences in *nad5*, *cob*, and *ccmf* genes discussed below, the mitogenomes of the six Euplotia species show synteny within the Euplotia, Hypotrichia, and Oligotrichia. Compared with hypotrichs and oligotrichs, the *nad5* gene of Euplotia spp. is inverted, and it is translocated in *E. vanleeuwenhoeki*. Apart from *E. raikovi* and *D. appendiculata*, the *cob* genes of the other four *Euplotes* are inverted in comparison with hypotrichs and oligotrichs. In addition, the position of the split *ccmf* gene (*ccmf_i*, *ccmf_ii*) varies within Euplotia. The two parts of *ccmf* gene are closely adjacent in *E. vanleeuwenhoeki*, *E. minuta*, *E. vannus*, and *E. crassus*. In addition, *ccmf_i* and *ccmf_ii* genes are collinear between *E. vanleeuwenhoeki* and the hypotrichs + oligotrichs but are inverted in the other three taxa. The *ccmf_i* and *ccmf_ii* genes are not closely adjacent to each other in *E. raikovi* and *D. appendiculata*. The *ccmf_i* gene is located at the 5′ end and *ccmf_ii* is located at the 3′ end. Among them, *ccmf_ii* shows synteny with that of *E. crassus*, *E. vannus*, and *E. minuta*, while *ccmf_i* is inverted. Finally, *rps4*, *cox1*, and *cox2* genes of *E. raikovi* are translocated within Euplotia (Fig. 2).

### Phylogenetic analyses

Phylogenetic analyses focusing on all aerobic ciliates with reported mitogenomes and a representative anaerobic ciliate were carried out, including nine subclasses of four classes of ciliates (Fig. 3). Based on concatenated mitochondrial proteins, seven subclasses are monophyletic; two of the clades, Armophorea (*Nyctotherus ovalis*) and Peritrichia (*Thuricola similis*), only included one species each (Fig. 3). Within the class Spirotrichea, the two *Pseudokeronopsis* isolates group with each other and fall into the clade of Hypotrichia. The two populations of *Euplotes crassus* cluster with *E. vannus*_new, forming a fully supported sister clade to *E. vannus*_old. In addition, *E. aediculatus* and *E. octocarinatus* occupy a basal position of the *Euplotes* clade, and *E. raikovi* falls outside of the assemblage *E. crassus* + *E. vannus* + *E. minuta* + *E. focardii* + *E. vanleeuwenhoeki*. Furthermore, *D. appendiculata* clusters with *Euplotes* taxa in the ML tree but groups with the Hypotrichia and Oligotrichia in the BI tree. Within the class Oligohymenophorea, the subclass Scuticociliatia groups with Hymenostomatia, forming a sister clade to Peniculia with high support. *Thuricola similis* falls outside of the three subclasses described above.

**Fig. 4:**
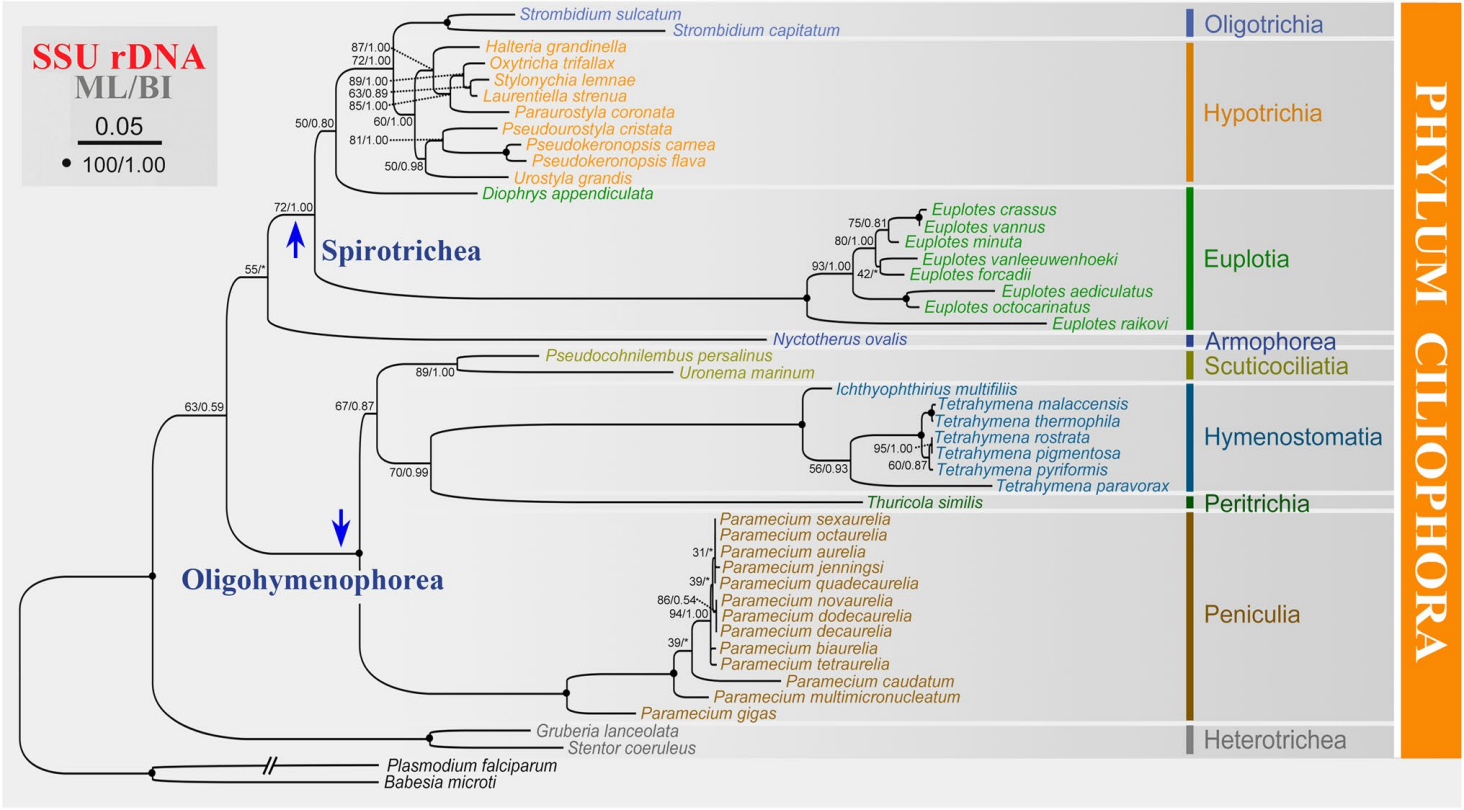
Maximum Likelihood (ML) tree based on SSU rDNA data. Numbers at the nodes represent the bootstrap values of ML and the posterior probability values of Bayesian analysis (BI). Asterisks (*) indicate disagreement between the ML and BI methods. The scale bar corresponds to five substitutions per 100 nucleotide positions

The mitochondrial tree and the SSU rDNA tree exhibit almost identical topology (Figs. 3, 4). The main differences are: (1) *D. appendiculata* clusters with the Hypotrichia and Oligotrichia, rather than with *Euplotes* spp.; (2) *Pseudourostyla*, *Urostyla*, and *Pseudokeronopsis* group together with low to high support; (3) *E. raikovi* falls outside of all the remaining *Euplotes* species with full support; (4) *T. similis* nests within the class Oligohymenophorea and clusters with the Hymenostomatia.

### Codon usage analyses

**Fig. 5:**
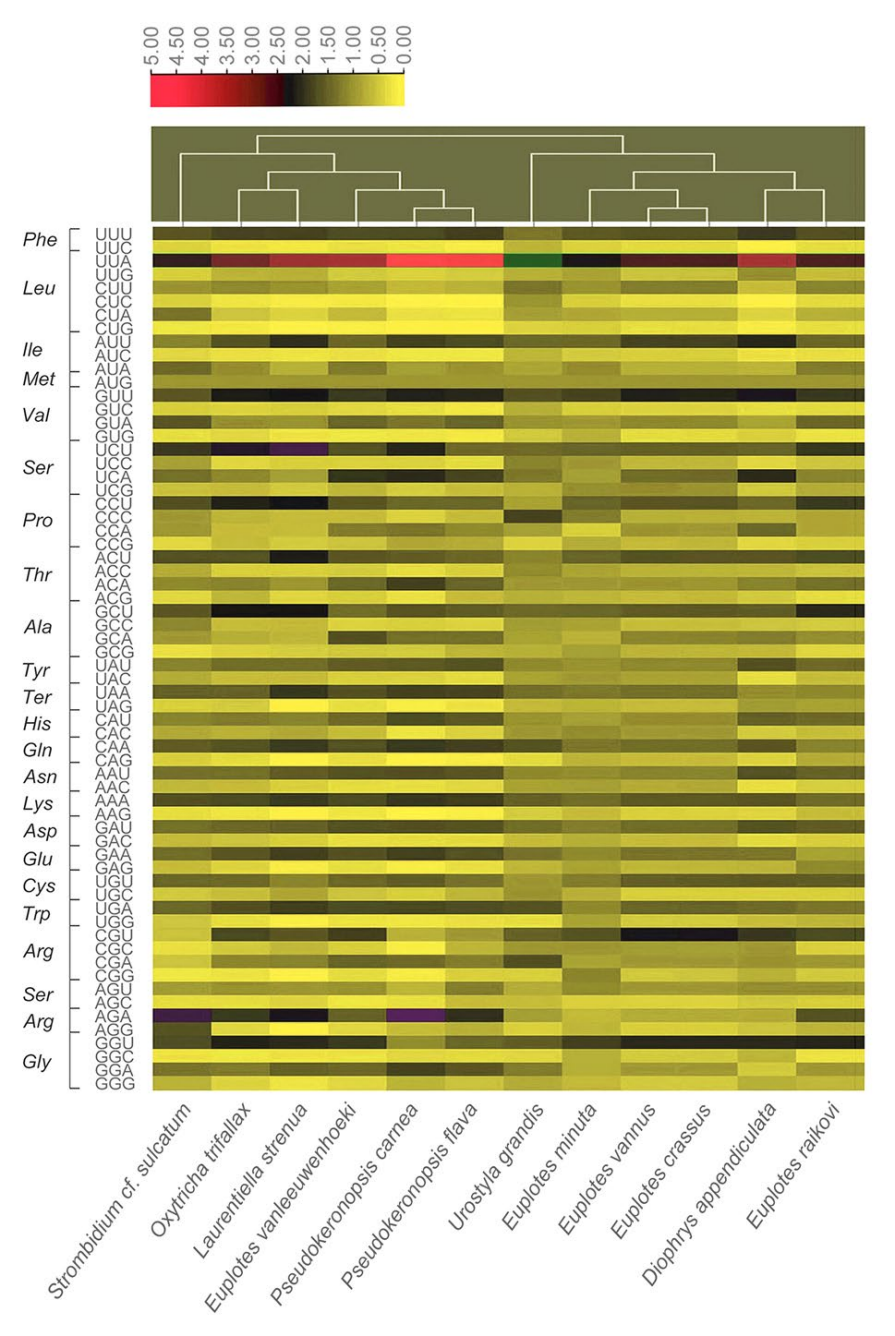
The heatmap of RSCU values of codons in mitogenomes of 12 Spirotrichea representatives. The scale bar represents RSCU value

In the mitogenomes of spirotrichs, the codon UGA is used to encode Trp in the mitochondrion, instead of its assignment as a stop codon in the standard genetic code (Supplementary Table S3). The mitochondrial protein-coding genes exhibit extensive codon usage bias (RSCU > 1) within Spirotrichea (Fig. 5). In *Urostyla grandis*, CCC and UGC are, respectively, the more commonly used codons to encode Pro and Cys. In the other species, CCU and UGU are, respectively, the more commonly used codons for Pro and Cys. In addition, His is more often encoded using the triplet CAC in *E. minuta*, while CAU is more commonly used in the remaining taxa. The codon GAG is used more often in *E. raikovi*, whereas it is used less often in the other species (Fig. 5; Supplementary Table S3).

**Fig. 6:**
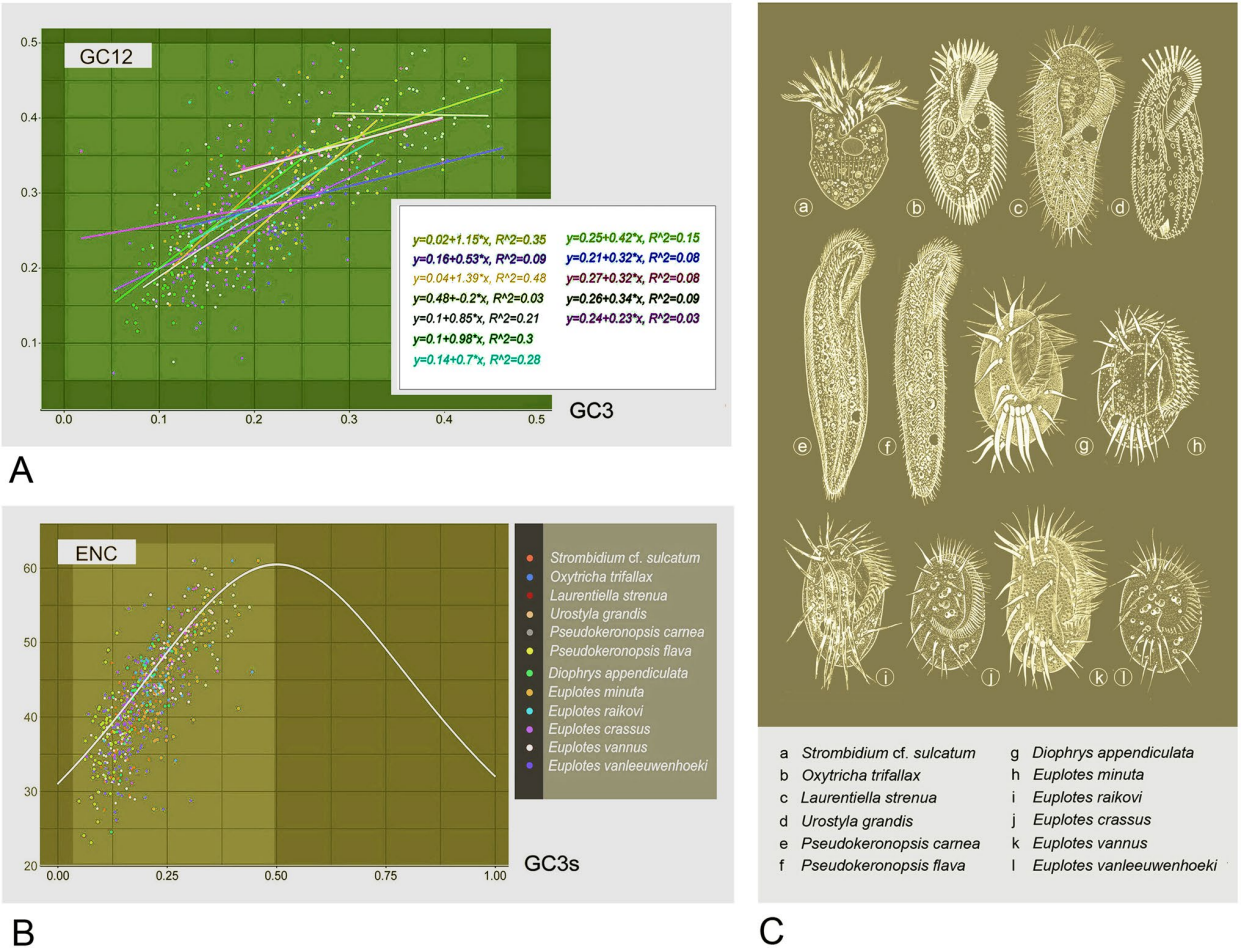
Codon usage bias in mitogenomes and morphological illustration of Spirotrichea. **A** Neutrality plot analysis of protein-coding genes in 12 mitogenomes. The lines represent regression lines. Equations defining each line are shown and include the slope. **B** ENC plot analysis of protein-coding genes in 12 mitogenomes. The line represents the standard curve derived from expected ENC. **C** The morphological figures of 12 representatives of Spirotrichea. Illustrations are original from authors’ group or from Augustin and Foissner (1992), Shin (1994), Song et al. (2009) and Zhang et al. (2022)

According to the neutrality plot, GC12 and GC3 exhibit linearity for all species with *P* < 0.01 based on *t* test (Fig. 6). ENC analyses show greater codon bias (ENC ≤ 35) for mitochondrial protein-coding genes in *O. trifallax*, *L. strenua*, *P. carnea*, and *P. flava*, when compared with the others (Fig. 6).

### nad2 proteins within Spirotrichea

**Fig. 7:**
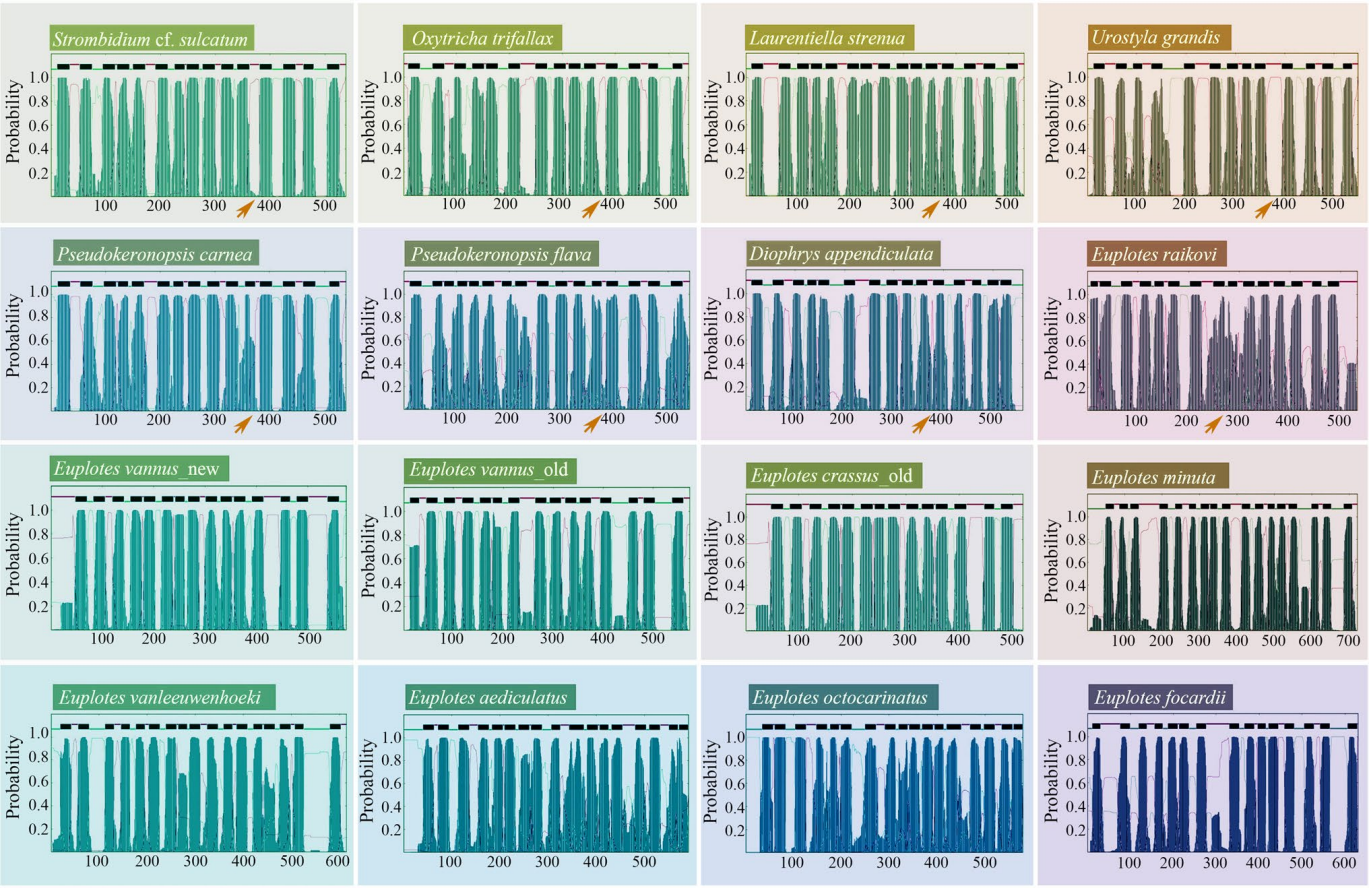
Transmembrane helix prediction of putative nad2 proteins for representatives of Spirotrichea ciliates. The *x*-axis shows amino acid position along the length of the protein and the *y*-axis shows transmembrane probability. The light green, purple, and dark green lines represent inside, outside, and transmembrane, respectively. The arrow indicates the split position of nad2_a and nad2_b

**Fig. 8:**
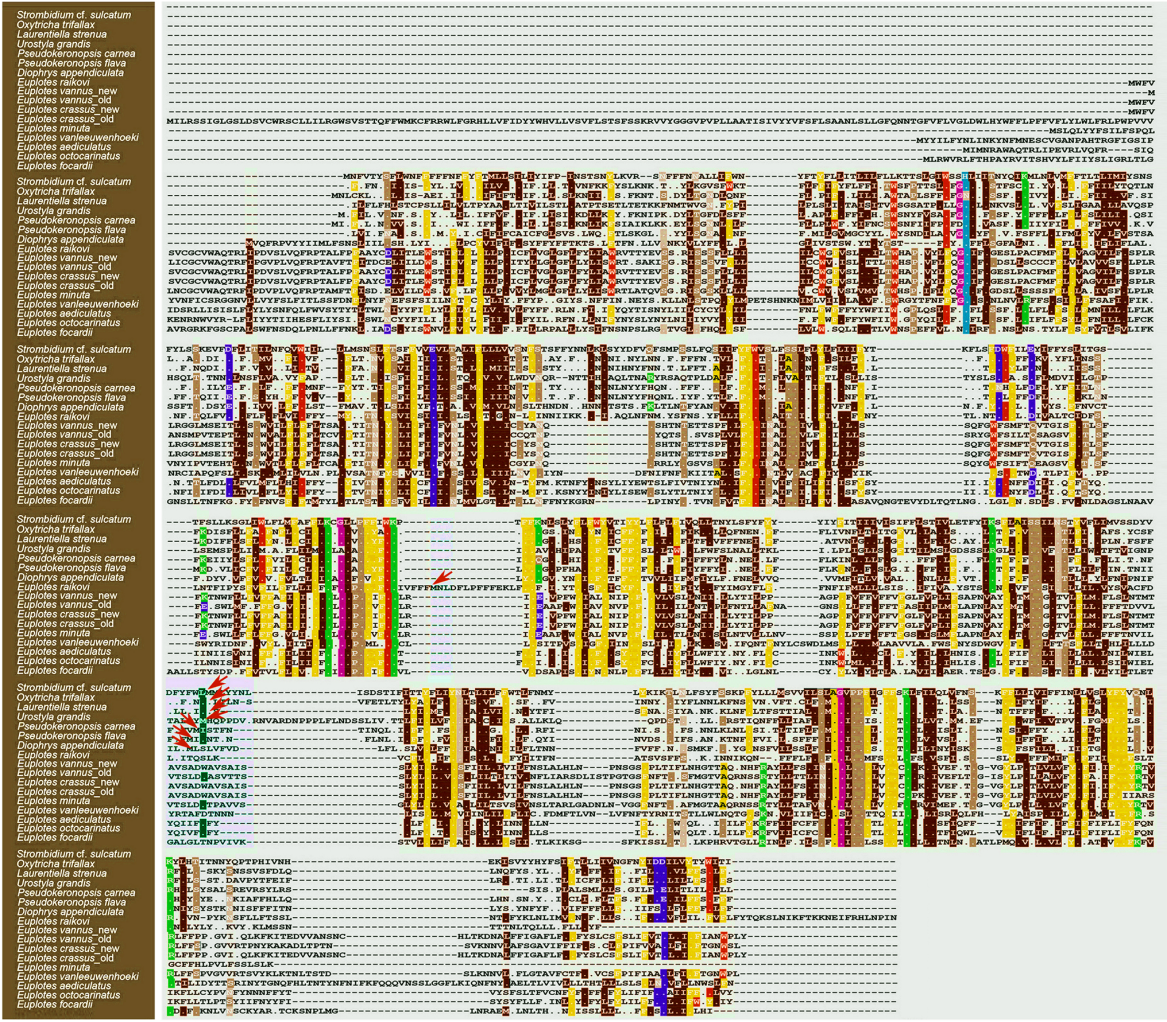
Sequence comparison of the nad2 proteins within Spirotrichea. Dashes represent sequence deletion or insertion. The dot indicates identical amino acid to that in the first sequence. Arrows show the split position of nad2_a and nad2_b

The number of transmembrane helices of nad2 proteins are similar within the Spirotrichea: 13–16 are detected in the hypotrichs, and 15 are detected oligotrichs; *D. appendiculata* possesses 16 helices, and *E. raikovi* possesses 15; the other *Euplotes* species with non-split nad2 proteins have 14–17 transmembrane helices (Fig. 7). The sequence alignment shows that nad2 proteins of spirotrichs are generally conserved except for N-terminal extensions of *Euplotes* spp. In addition, the split position of nad2_a and nad2_b is basically the same in the alignment for *S*. cf. *sulcatum*, *O. trifallax*, *L. strenua*, *U. gandis*, *P. carnea*, *P. flava*, and *D. appendiculata*. Regarding *E. raikovi*, although the split position differs from the other species, the front and back positions remain conserved (Fig. 8). Finally, the HHpred results indicate that the protein sequences in four *Euplotes* species can be mapped to a nad2 protein (*Brassica oleracea*). The other species matched the monovalent Na^+^/H^+^ antiporter subunit D protein of *Dietzia* sp., which is the inactivated form of respiratory chain complex I and is assigned the molecular function term NADH dehydrogenase in Gene Ontology analysis (Roberts and Hirst 2012) (Table 3), which is shared with nad2.

**Table 3:** HHpred results of nad2 proteins within Spirotrichea

Species	Length (aa)	Top hit	Aligned cols	Probability (%)	*e*-value
*Strombidium* cf. *sulcatum*	538 (369, 169)	*Dietzia* sp. Monovalent Na^+^/H^+^ antiporter subunit D	418 aa	100	1e−47
*Oxytricha trifallax*	538 (371, 167)	*Dietzia* sp. Monovalent Na^+^/H^+^ antiporter subunit D	363 aa	100	2.3e−47
*Laurentiella strenua*	529 (373, 156)	*Dietzia* sp. Monovalent Na^+^/H^+^ antiporter subunit D	363 aa	100	1.7e−44
*Urostyla grandis*	548 (369, 179)	*Dietzia* sp. Monovalent Na^+^/H^+^ antiporter subunit D	362 aa	100	7.3e−48
*Pseudokeronopsis carnea*	539 (371, 168)	*Dietzia* sp. Monovalent Na^+^/H^+^ antiporter subunit D	363 aa	100	3.1e−46
*Pseudokeronopsis flava*	540 (371, 169)	*Dietzia* sp. Monovalent Na^+^/H^+^ antiporter subunit D	418 aa	100	7e−47
*Diophrys appendiculata*	560 (369, 191)	*Dietzia* sp. Monovalent Na^+^/H^+^ antiporter subunit D	420 aa	100	1.1e−46
*Euplotes raikovi*	563 (287, 246)	*Brassica oleracea* nad2	367 aa	100	1.2e−41
*Euplotes vannus*_new	574	*Brassica oleracea* nad2	399 aa	100	4.2e−46
*Euplotes vannus*_old	573	*Brassica oleracea* nad2	427 aa	100	3.1e−46
*Euplotes crassus*_new	574	*Brassica oleracea* nad2	398 aa	100	2.4e−46
*Euplotes crassus*_old	519	*Brassica oleracea* nad2	425 aa	100	3.6e−43
*Euplotes minuta*	721	*Brassica oleracea* nad2	401 aa	100	2.3e−42
*Euplotes vanleeuwenhoeki*	614	*Dietzia* sp. Monovalent Na^+^/H^+^ antiporter subunit D	358 aa	100	3.6e−43
*Euplotes aediculatus*	591	*Dietzia* sp. Monovalent Na^+^/H^+^ antiporter subunit D	361 aa	100	1.7e−43
*Euplotes octocarinatus*	579	*Brassica oleracea* nad2	406 aa	100	1.1e−41
*Euplotes focardii*	630	*Dietzia* sp. Monovalent Na^+^/H^+^ antiporter subunit D	362 aa	100	3e−42

The numbers in brackets indicate protein lengths of nad2_a and nad2_b, respectively

## Discussion

### Split and evolution of the *nad2* gene

According to Swart et al. (2011) and Zhang et al. (2021), the mitochondrial *nad2* gene is fragmented into two parts (*nad2_a*, *nad2_b*) in the Hypotrichia and Oligotrichia. All of our analyses of transmembrane helix structure, protein alignment, and HHpred prediction reveal that the situation of the *nad2* gene in Euplotia is complex: *nad2* is split in *D. appendiculata* and *E. raikovi*, while it is non-split in the remaining *Euplotes* spp. (Figs. 7, 8; Table 3).

For the anaerobic Armophorea ciliates with reported mitogenomes (except for *Metopus laminarius* lacking *nad2*), *nad2* gene is split in *Nyctotherus ovalis* and *Heterometopus* sp. (De Graaf et al. 2011; Lewis et al. 2020; Rotterová et al. 2020). For mitogenomes of *M. contortus* and an unnamed metopid species (metopid sp. SK), another split part of the *nad2* gene is also found in the adjacent position of the originally annotated *nad2* part, based on blastp searches in this work. In addition, *nad2* gene is also split in the Oligohymenophorea (Johri et al. 2019; Swart et al. 2011). However, it is non-split in two reported heterotrichs (*Stentor coeruleus* and *Gruberia lanceolata*) (Park and Min 2019; Slabodnick et al. 2017). Given that the class Heterotrichea typically represents a primitive group and occupies a basal position in the phylogenetic tree of ciliates (Chi et al. 2021; Gao et al. 2016), we speculate that the non-split status of *nad2* gene is an ancient feature. However, in this study, it is challenging to determine whether the *nad2* gene in *Euplotes* spp. (except for *E. raikovi*) retains this ancient characteristic or if it fused again from separate *nad2_a* and *nad2_b* genes during evolution. Further evidence (e.g., by targeting other species of *Euplotes*) is needed to draw a robust conclusion. In addition, the *nad2_a* and *nad2_b* genes are adjacent to each other in mitogenomes of oligotrichs, hypotrichs, *D. appendiculata*, *E. raikovi*, *Heterometopus* sp., *M. contortus*, and an unnamed metopid species (metopid sp. SK), whereas they are usually separated by other genes in mitogenomes of the Oligohymenophorea and *N. ovalis* (Huang et al. 2021b; Lewis et al. 2020; Rotterová et al. 2020; Swart et al. 2011; Zhang et al. 2021). We speculate that this might be due to a genomic rearrangement or gene transposition that has further separated the two *nad2* parts.

### Small subunit ribosomal proteins encoded in the mitogenome

According to Zhang et al. (2021), spirotrichs possess the largest and most gene-rich mitogenomes among ciliates, which is consistent with our analyses of gene component (Fig. 3). In this study, the mitogenome of one representative anaerobic ciliate (class Armophorea) and all available mitogenomes of aerobic ciliates (class Heterotrichea, Oligohymenophorea and Spirotrichea) are used for gene component analyses. Among them, the hydrogenosome of the anaerobic ciliate shows the greatest reduction in gene composition, which is the result of anaerobic evolution of the mitochondrion and is accordant with previous studies (De Graaf et al. 2011; Lewis et al. 2020). For the remaining three classes of ciliates, the complement of small subunit ribosomal protein genes (rps) is more complicated, despite the nearly identical categorization of mitochondrial genes.

Research on mitogenomes indicates the presence of 12 rps genes in the mitochondria of protists, including *rps1–4*, *rps7–8*, *rps10–14*, and *rps19* (Gray et al. 2004). Except for *rps1* and *rps11* genes, the small ribosomal proteins of ciliates are fairly complete (Fig. 3). Furthermore, there is a trend for an increased number of rps genes in the more recently branching ciliate classes (Heterotrichea: four or six genes, Oligohymenophorea: five genes, and Spirotrichea: 7–10 genes) (Gao et al. 2016), with the most complete complement of rps genes in the mitogenomes of oligotrichs and hypotrichs. Among the ten rps genes of ciliates, *rps12*, *rps14*, *rps3*, *rps19*, and *rps13* are conserved and present in almost all aerobic mitochondria of ciliates. Notably, *rps10* gene is specific to spirotrichs, *rps2* gene is unique to oligotrichs and hypotrichs, *rps4* gene is absent only in Oligohymenophorea, and *rps13* gene is absent in heterotrichs. In addition, when combined with phylogenetic results, the *rps8* gene appears to have been lost during the evolution of *Euplotes* taxa.

Regarding the absent genes in the mitogenome of ciliates, we propose four possibilities: (1) these genes are genuinely absent from the mitogenome due to their endosymbiotic gene transfer to the nucleus, with subsequent import of the proteins into the mitochondria (Gray and Archibald 2012; Roger et al. 2017); (2) low selective pressure and rapid evolution of these genes have resulted in the low sequence similarities when compared to their orthologs (Barth and Berendonk 2011; Chen 2015); (3) failure to identify these genes could be the result of sequencing or assembly errors; and (4) poor synteny of these genes between congeners and related species has led to their exclusion from sequencing or assembly. Which of these possibilities describes the absence of each gene may differ based on the gene and the species of ciliate studied.

### Phylogenetic analysis

Studies on the genus *Diophrys* (belonging to the family Uronychidae) show that it shares common morphological characteristics with euplotids, such as highly developed transverse cirri, marginal and caudal cirri, a sculptured surface, and well-developed adoral zone of membranelles (Shao et al. 2020; Song et al. 2009). Based on these features, *Diophrys* was assigned to the subclass Euplotia and closely related to the other four families in Euplotida (Euplotidae, Certesiidae, Aspidiscidae, and Gastrocirrhidae) (Dong et al. 2022; Shao et al. 2020), which is consistent with some phylogenetic analyses based on rDNA markers (SSU rDNA, ITS1-5.8S-ITS2 and LSU rDNA (large subunit ribosomal RNA gene)) (Lian et al. 2023; Yi et al. 2009). However, some molecular evidence shows a divergent phylogenetic position of *Diophrys*. For example, the SSU rDNA and ITS1-5.8S-ITS2 trees indicate that it is sister to the group of Euplotidae + Aspidiscidae + Hypotrichia (Huang et al. 2012). Furthermore, the family Uronychidae forms a sister clade to the assemblage comprising Certesiidae + Euplotidae + Aspidiscidae + Hypotrichia + Oligotrichia + Phacodiniidia according to phylogenetic analyses inferred from *cox1* data (Lian et al. 2023). Yi et al. (2012) showed that some taxa of the polyphyletic genus *Diophrys* have close relationships with hypotrichs based on *alpha-tubulin*. Consistent with the result of Yi et al. (2012), *D. appendiculata* has a closer relationship with hypotrichs and oligotrichs than to *Euplotes* in our SSU rDNA tree (Fig. 4). However, the AU test does not reject the monophyly of *D. appendiculata* and *Euplotes* spp. (*P* = 0.665). Although *D. appendiculata* nests within Euplotida in phylogenetic trees based on mitochondrial data, the support values are not high (Fig. 3). All of the above implies that the phylogenetic position of *Diophrys* is unstable.

To improve our assessment of the phylogenetic position of *D. appendiculata*, the mitogenome information is also compared here. Specifically, the mitochondrial gene components observed in *D. appendiculata*, including *rps7*, split *nad2*, and split *rps3* genes, resemble those in hypotrichs and oligotrichs instead of *Euplotes* species (Figs. 2, 3). In addition, *trnL* gene is found in *D. appendiculata*, *S.* cf. *sulcatum*, *O. trifallax*, and *Laurentiella strenua*, but absent in other Spirotrichea taxa (Table 2). Furthermore, regarding gene collinearity, except for inversions of *ccmf_ii* and *nad5* genes, the remaining mitochondrial genes of *D. appendiculata* display conserved synteny with the hypotrichs and oligotrichs, especially the identical collinearity with *O. trifallax*, *L. strenua,* and *U. grandis* (Fig. 2). However, *D. appendiculata* also exhibits some similar mitogenome features with *Euplotes*, such as the absence of *rps2* and the gene arrangement of *nad5* and *ccmf* genes. Consequently, we suspect that *Diophrys* may represent an intermediate group between the Euplotia and Hypotrichia + Oligotrichia. However, only one mitogenome is available, so it is too early to change the taxonomic status of *Diophrys*. More mitochondrial and molecular evidence is needed to obtain a more robust conclusion.

### Codon usage bias

Previous studies on codon usage have indicated that the use of synonymous triplets may arise from a dynamic interplay between mutation (nucleotide biases produced by point mutations, or in the rates or repair of point mutations), natural selection, and genetic drift, and this can contribute to distinct codon usage patterns and genome evolution (Liu 2012; Mazumdar et al. 2017; Parvathy et al. 2022). In this study, the heatmap of RSCU shows evident codon bias in the mitogenomes of Spirotrichea species (Fig. 5). In addition, the ENC plot indicates that the actual ENC values are lower than expected values for most protein-coding genes (Fig. 6), further supporting the asymmetric usage of synonymous codons. Furthermore, the more commonly used synonymous codons end in either A or T (Fig. 5; Supplementary Table S3). This is consistent with the low G+C content of the mitogenome (Table 1), given that the tendency of nucleotide bases of codons is likely influenced by the genomic G+C content (Iriarte et al. 2021).

To clarify the factors shaping codon usage, neutrality and ENC plots were analyzed. According to previous research, codon usage bias is mainly influenced by mutation pressure when the slope of the regression line in neutrality plots is close to 1, while it is due to selection pressure if the slope is closer to zero (Huang et al. 2021a; Parvathy et al. 2022; Yu et al. 2021). In this study, the slopes of regression lines near to 1 in the mitochondria of *S.* cf. *sulcatum*, *O. trifallax*, *L. strenua*, *P. carnea*, *P. flava*, and *D. appendiculata* suggest that the codon biases seen in these species are mainly influenced by mutations at the DNA level (Fig. 6), i.e., genome compositional bias (Mazumdar et al. 2017; Shabalina et al. 2013; Wang and Hickey 2007). Previous studies have also shown that the codon usage bias is more likely affected by mutation if genes distribute near the standard curve based on ENC plot analyses (Huang et al. 2021a; Liang et al. 2019; Liu et al. 2004). In this study, the actual ENC values of protein-coding genes in the species listed above are located around the standard curve, further supporting mutation as a main influence on codon usage bias (Fig. 6; Supplementary Table S4). In contrast, natural selection at the translation level likely plays the dominant role in codon variation in the mitogenomes of the remaining taxa, as indicated by the neutrality analyses (Fig. 6). However, the ENC plot shows that most actual ENC numbers situate near the standard line, implying that not only natural selection but also mutation may contribute to their codon bias (Figs. 5, 6; Supplementary Table S4).

In conclusion, codon usage bias in the mitogenomes of oligotrichs, hypotrichs (expect for *U. grandis*), and *D. appendiculata* with low mitogenomic G+C content (18.84–28.32%, average: 23.70%) may be due to mutation. In contrast, the codon bias might be affected by both genome mutation and natural selection on the encoded protein products for mitochondria in *Euplotes* spp. and *U. grandis* which show a high G+C content (25.09–38.88%, average: 32.59%) (Figs. 5, 6; Table 1). This differs from the conclusions obtained in Huang et al. (2021a) for *Euplotes* species that showed mutations as the main influence of codon usage bias in *Euplotes* mitogenomes. Regarding this controversy, we suggest that it may be due to the differences in the analysis method used. Specifically, in this study, the data were separately analyzed for each *Euplotes* isolate, whereas all protein-coding genes were combined together for codon bias analyses in Huang et al. (2021a). When all protein-coding genes of the five *Euplotes* species were combined for neutrality plot and regression analysis, we obtained a similar slope of regression line (0.57) with the slope result (0.549) in Huang et al. (2021a), supporting our hypothesis above.

## Supplementary Information

Below is the link to the electronic supplementary material.

Supplementary file1 (DOC 1553 KB)

## Data Availability

The mitogenome data and annotation information are deposited in NCBI GenBank database with the accession numbers as follows: *Pseudokeronopsis carnea* (PP853579), *Pseudokeronopsis flava* (PP853578), *Diophrys appendiculata* (PP808730), *Euplotes vannus* (PP853575), *Euplotes crassus* (PP853576), *Euplotes raikovi* (PP853577).
