# Supplementary material for: Novel findings on the mitochondria in ciliates, with description of mitochondrial genomes of six representatives
Source: Mar Life Sci Technol. 2024 Sep 23;7(1):79–95. doi: 10.1007/s42995-024-00249-7 (PMC11871222; doi:10.1007/s42995-024-00249-7)
Supplement: Supplementary file 1 — Supplementary file1 (DOC 1553 KB) [file 42995_2024_249_MOESM1_ESM.doc]

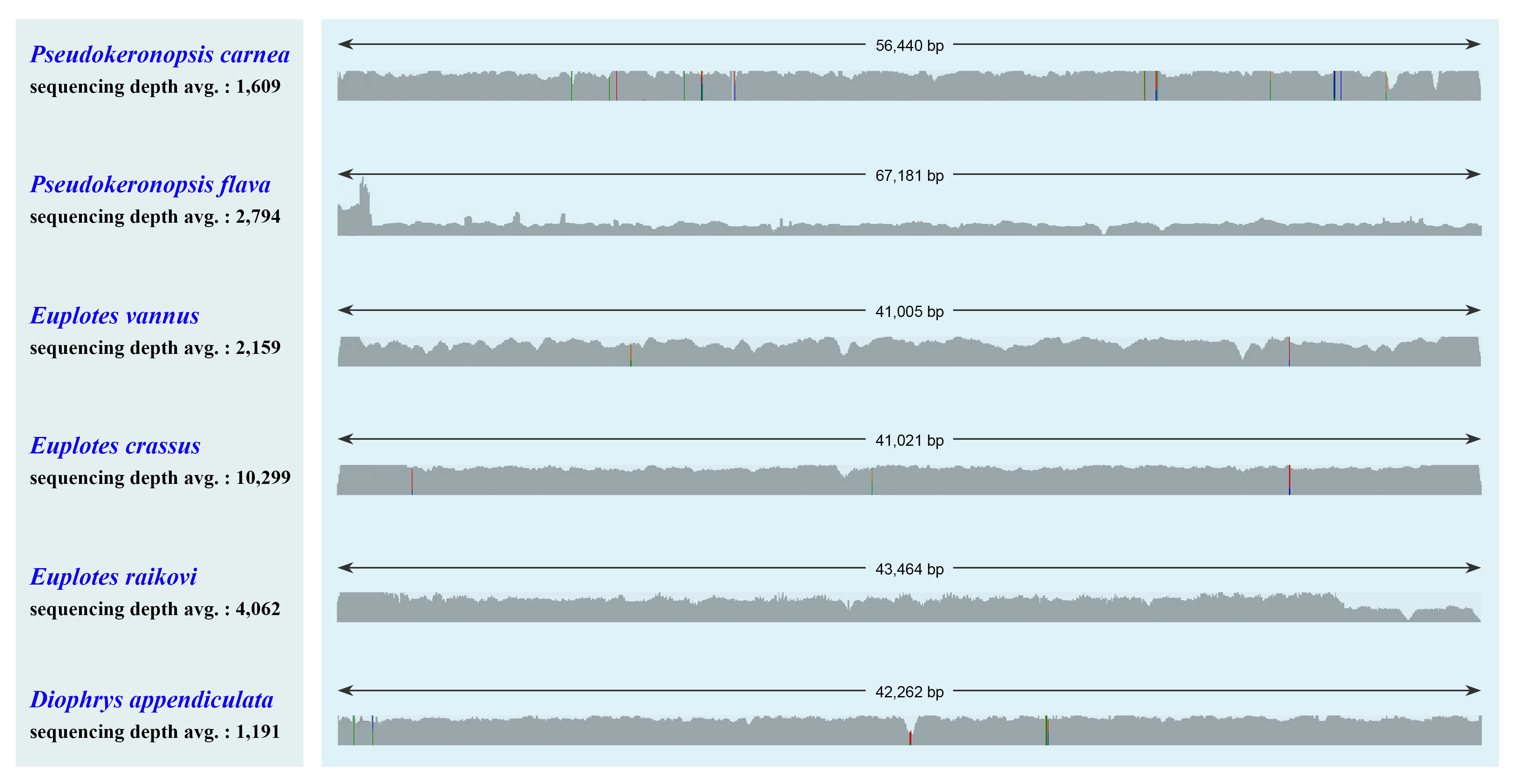
 **Fig.S1** Sequencing depth distribution of six new mitogenomes.

**Table S1** PCR Primers for mitogenome assemblies

| Species | Fragments | Primers | Sequences (5′–3′) |
| --- | --- | --- | --- |
| *Pseudokeronopsis carnea* | PCR_1 | 379_F1 | GAGGTGTTGTTGGGATTTG |
|  |  | 1480_R2 | TCGCTTCGTTAATTAGAGG |
|  | PCR_2 | 319_F1 | TTGGGGGACAACCAGGAACG |
|  |  | 1896_R1 | TACGGTTGATTGCCGAAAGC |
|  | PCR_3 | 473_F2 | ATGAGCTTTCGGCAATCAACCG |
|  |  | 1512_R1 | TGCTTTCCGCATTTACCTCT |
|  | PCR_4 | 581_F2 | CCAGTAGATCCAGATAATG |
|  |  | 1688_R2 | TGGCTCACAAAAACCTACAT |
| *Euplotes crassus* | PCR_1 | 295_F1 | CCGAGTGGACAAAGGCGGTAGAC |
|  |  | 1539_R1 | AGCATTTTACCGCTCCACTTACG |
|  | PCR_2 | 345_F1 | GGGGGGGTTGATGAGTTTGAGAC |
|  |  | 1876_R1 | CATAAAGTGCGGATGGGTGGC |
| *Euplotes raikovi* | PCR_1 | 581_F2 | ACGAAAGAAGTCTGGTGTAG |
|  |  | 1938_R1 | AGGTTAGAAATGGTGAAAGG |
|  | PCR_2 | 57_F1 | CTAGTCTGTTTACCTTTAACCGC |
|  |  | 607_R2 | GCTCAAATAATAACTGTGTAAGG |

**Table S2** GenBank accession numbers of mitogenome and SSU rDNA data used for phylogenetic analyses in present study. Newly characterized mitogenomes are in bold

| **Species** | **Accession No.** | | **Species** | **Accession No.** | |
| --- | --- | --- | --- | --- | --- |
|  | **Mitogenome** | **SSU rDNA** |  | **Mitogenome** | **SSU rDNA** |
| *Babesia microti* | NC_034637 | LC005772 | *Paramecium jenningsi* | * | LT628492 |
| *Diophrys appendiculata* | **PP808730** | AY004773 | *Paramecium multimicronucleatum* | * | LT628494 |
| *Euplotes aediculatus* | MT665958 | KX867114 | *Paramecium novaurelia* | * | LN869961 |
| *Euplotes crassus*_new | **PP853576** | - | *Paramecium octaurelia* | * | MG009442 |
| *Euplotes crassus*_old | GQ903131 | AY007439 | *Paramecium quadecaurelia* | * | OQ249519 |
| *Euplotes forcadii* | SRR3929761 | MW369534 | *Paramecium sexaurelia* | * | OQ249522 |
| *Euplotes minuta* | GQ903130 | FJ876977 | *Paramecium tetraurelia* | * | KY852452 |
| *Euplotes octocarinatus* | SRR2474557 | MK411264 | *Plasmodium falciparum* | M76611 | JQ627152 |
| *Euplotes raikovi* | **PP853577** | MN783334 | *Pseudokeronopsis carnea* | **PP853579** | KU715982 |
| *Euplotes vanleeuwenhoeki* | MK889230 | OU070011 | *Pseudourostyla cristata* | MH888186 | JN714476 |
| *Euplotes vannus*_new | **PP853575** | - | *Pseudokeronopsis flava* | **PP853578** | FJ598608 |
| *Euplotes vannus*_old | MT665959 | AY004772 | *Pseudocohnilembus persalinus* | MH608212 | AY551906 |
| *Halteria grandinella* | MT471317 | MF002432 | *Stentor coeruleus* | MPUH01000652 | KY855543 |
| *Ichthyophthirius multifiliis* | JN227086 | MN372056 | *Strombidium capitatum* | - | KP260510 |
| *Gruberia lanceolate* | MK301177 | MH024390 | *Strombidium* cf. *sulcatum* | MT471316 | FJ377546 |
| *Laurentiella strenua* | KX529838 | JX893368 | *Strombidium* sp. | MT471315 | - |
| *Nyctotherus ovalis* | GU057832 | AY007456 | *Stylonychia lemnae* | KX524144 | AM233908 |
| *Oxytricha trifallax* | JN383843 | KC193240 | *Tetrahymena malaccensis* | DQ927303 | M26360 |
| *Paraurostyla coronata* | - | KU715982 | *Tetrahymena paravorax* | DQ927304 | EF070253 |
| *Paraurostyla* sp. | KX524143 | - | *Tetrahymena pigmentosa* | DQ927305 | MH051923 |
| *Paramecium aurelia* | NC001324 | KY855583 | *Tetrahymena pyriformis* | AF160864 | MH051925 |
| *Paramecium biaurelia* | * | MH822861 | *Tetrahymena rostrata* | MN025427 | AF364042 |
| *Paramecium caudatum* | FN424190 | MG589314 | *Tetrahymena thermophila* | AF396436 | X56165 |
| *Paramecium decaurelia* | * | FJ003994 | *Thuricola similis* | MW221262 | MW208818 |
| *Paramecium dodecaurelia* | * | FJ004000 | *Uronema marinum* | MG272262 | GQ259749 |
| *Paramecium gigaas* | MT622823 | OQ249518 | *Urostyla grandis* | KX494929 | KP681648 |

Note: “*” indicates the mitogenome data of ten *Paramecium* species which are not available on the NCBI database but can be accessed on Zenodo (<https://doi.org/10.5281/zenodo.2539699>. “-” indicates that no data were used in this study

**Table S3** The RSCU (relative synonymous codon usage) values of mitochondrial proteins within Spirotrichea

| **amino acids** | **codons** | ***Stombidium* cf. *sulcatum*** | ***Oxytricha trifallax*** | ***Laurentiella strenua*** | ***Urostyla grandis*** | ***Pseudokeronopsis carnea*** | ***Pseudokeronopsis flava*** | ***Diophrys appendiculata*** | ***Euplotes vannus*** | ***Euplotes crassus*** | ***Euplotes minuta*** | ***Euplotes vanleeuwenhoeki*** | ***Euplotes raikovi*** |
| --- | --- | --- | --- | --- | --- | --- | --- | --- | --- | --- | --- | --- | --- |
| **Phe** | UUU | 1.60 | 1.79 | 1.81 | 1.30 | 1.76 | 1.83 | 1.89 | 1.66 | 1.66 | 1.59 | 1.73 | 1.68 |
|  | UUC | 0.40 | 0.21 | 0.19 | 0.70 | 0.24 | 0.17 | 0.11 | 0.34 | 0.34 | 0.41 | 0.27 | 0.32 |
| **Leu** | UUA | 2.58 | 3.19 | 3.58 | 1.60 | 4.53 | 4.42 | 3.68 | 2.82 | 2.82 | 2.38 | 3.66 | 2.83 |
|  | UUG | 0.43 | 0.70 | 0.74 | 0.75 | 0.41 | 0.47 | 1.07 | 0.54 | 0.56 | 0.85 | 0.49 | 0.6 |
|  | CUU | 1.01 | 1.14 | 1.04 | 1.32 | 0.64 | 0.72 | 0.61 | 1.26 | 1.26 | 1.04 | 0.88 | 1.18 |
|  | CUC | 0.42 | 0.24 | 0.18 | 1.02 | 0.10 | 0.08 | 0.09 | 0.29 | 0.29 | 0.45 | 0.2 | 0.34 |
|  | CUA | 1.33 | 0.54 | 0.38 | 0.90 | 0.27 | 0.26 | 0.38 | 0.77 | 0.77 | 0.82 | 0.63 | 0.76 |
|  | CUG | 0.23 | 0.19 | 0.09 | 0.40 | 0.05 | 0.05 | 0.17 | 0.31 | 0.3 | 0.45 | 0.14 | 0.3 |
| **Ile** | AUU | 1.21 | 1.67 | 1.99 | 1.47 | 1.85 | 1.76 | 2.09 | 1.79 | 1.79 | 1.45 | 1.45 | 1.45 |
|  | AUC | 0.36 | 0.27 | 0.26 | 0.69 | 0.23 | 0.23 | 0.21 | 0.44 | 0.45 | 0.48 | 0.27 | 0.29 |
|  | AUA | 1.43 | 1.06 | 0.75 | 0.84 | 0.92 | 1.01 | 0.7 | 0.76 | 0.76 | 1.06 | 1.28 | 1.27 |
| **Met** | AUG | 1.00 | 1.00 | 1.00 | 1.00 | 1.00 | 1 | 1 | 1 | 1 | 1 | 1 | 1 |
| **Val** | GUU | 1.61 | 2.19 | 2.30 | 1.69 | 2.15 | 2.09 | 2.39 | 2.16 | 2.13 | 1.82 | 1.93 | 1.94 |
|  | GUC | 0.45 | 0.38 | 0.46 | 0.76 | 0.30 | 0.2 | 0.3 | 0.38 | 0.39 | 0.46 | 0.36 | 0.35 |
|  | GUA | 1.63 | 1.07 | 1.03 | 1.03 | 1.31 | 1.51 | 0.89 | 1.13 | 1.13 | 0.98 | 1.48 | 1.45 |
|  | GUG | 0.31 | 0.36 | 0.21 | 0.52 | 0.25 | 0.19 | 0.42 | 0.33 | 0.34 | 0.74 | 0.23 | 0.26 |
| **Ser** | UCU | 1.89 | 2.51 | 2.76 | 1.41 | 2.09 | 1.43 | 1.5 | 1.52 | 1.52 | 1.51 | 1.72 | 1.95 |
|  | UCC | 0.91 | 0.37 | 0.44 | 1.19 | 0.41 | 0.35 | 0.37 | 0.65 | 0.65 | 0.97 | 0.41 | 0.53 |
|  | UCA | 1.37 | 1.14 | 0.92 | 1.29 | 2.03 | 1.8 | 2.03 | 1.4 | 1.42 | 0.92 | 1.96 | 1.19 |
|  | UCG | 0.56 | 0.62 | 0.49 | 0.73 | 0.48 | 0.58 | 0.52 | 1.06 | 1.04 | 1.07 | 0.66 | 0.79 |
| **Pro** | CCU | 1.69 | 2.13 | 2.31 | 0.88 | 1.47 | 1.48 | 1.46 | 1.61 | 1.65 | 1.51 | 1.63 | 1.9 |
|  | CCC | 0.96 | 0.69 | 0.62 | 1.81 | 0.41 | 0.59 | 0.7 | 0.75 | 0.73 | 1.26 | 0.6 | 0.83 |
|  | CCA | 0.99 | 0.61 | 0.58 | 0.92 | 1.30 | 1.13 | 1.47 | 1.01 | 0.97 | 0.45 | 1.22 | 0.83 |
|  | CCG | 0.36 | 0.58 | 0.49 | 0.39 | 0.82 | 0.8 | 0.36 | 0.63 | 0.66 | 0.79 | 0.55 | 0.44 |
| **Thr** | ACU | 1.71 | 1.72 | 2.17 | 1.17 | 1.57 | 1.45 | 1.65 | 1.66 | 1.65 | 1.51 | 1.6 | 1.74 |
|  | ACC | 0.80 | 0.43 | 0.46 | 1.10 | 0.32 | 0.44 | 0.66 | 0.72 | 0.71 | 0.85 | 0.48 | 0.56 |
|  | ACA | 1.13 | 1.18 | 1.00 | 0.97 | 1.84 | 1.51 | 1.2 | 1.01 | 1 | 0.96 | 1.47 | 1.38 |
|  | ACG | 0.36 | 0.67 | 0.37 | 0.76 | 0.27 | 0.6 | 0.5 | 0.62 | 0.64 | 0.68 | 0.45 | 0.32 |
| **Ala** | GCU | 1.61 | 2.24 | 2.24 | 1.43 | 1.65 | 1.55 | 1.55 | 1.56 | 1.58 | 1.49 | 1.39 | 2.05 |
|  | GCC | 1.15 | 0.59 | 0.63 | 0.94 | 0.46 | 0.38 | 0.55 | 0.58 | 0.57 | 0.87 | 0.39 | 0.51 |
|  | GCA | 0.97 | 0.77 | 0.66 | 0.88 | 1.38 | 1.38 | 1.26 | 1.16 | 1.17 | 0.72 | 1.7 | 1.08 |
|  | GCG | 0.27 | 0.39 | 0.47 | 0.75 | 0.50 | 0.69 | 0.64 | 0.7 | 0.68 | 0.92 | 0.52 | 0.37 |
| **Tyr** | UAU | 1.21 | 1.40 | 1.39 | 1.11 | 1.59 | 1.66 | 1.68 | 1.14 | 1.14 | 1.04 | 1.56 | 1.41 |
|  | UAC | 0.79 | 0.60 | 0.61 | 0.89 | 0.41 | 0.34 | 0.32 | 0.86 | 0.86 | 0.96 | 0.44 | 0.59 |
| **Ter** | UAA | 1.54 | 1.52 | 1.95 | 1.38 | 1.85 | 1.79 | 1.07 | 1.38 | 1.38 | 1.29 | 1.69 | 1.12 |
|  | UAG | 0.46 | 0.48 | 0.05 | 0.63 | 0.15 | 0.21 | 0.93 | 0.62 | 0.62 | 0.71 | 0.31 | 0.88 |
| **His** | CAU | 1.19 | 1.27 | 1.19 | 1.03 | 1.74 | 1.59 | 1.52 | 1.05 | 1.05 | 0.91 | 1.41 | 1.43 |
|  | CAC | 0.81 | 0.73 | 0.81 | 0.97 | 0.26 | 0.41 | 0.48 | 0.95 | 0.95 | 1.09 | 0.59 | 0.57 |
| **Gln** | CAA | 1.56 | 1.67 | 1.90 | 1.66 | 1.93 | 1.83 | 1.62 | 1.39 | 1.4 | 1.3 | 1.72 | 1.2 |
|  | CAG | 0.44 | 0.33 | 0.10 | 0.34 | 0.07 | 0.17 | 0.38 | 0.61 | 0.6 | 0.7 | 0.28 | 0.8 |
| **Asn** | AAU | 1.35 | 1.38 | 1.56 | 1.12 | 1.69 | 1.64 | 1.71 | 1.2 | 1.19 | 1.09 | 1.67 | 1.53 |
|  | AAC | 0.65 | 0.62 | 0.44 | 0.88 | 0.31 | 0.36 | 0.29 | 0.8 | 0.81 | 0.91 | 0.33 | 0.47 |
| **Lys** | AAA | 1.69 | 1.75 | 1.91 | 1.53 | 1.94 | 1.86 | 1.63 | 1.58 | 1.59 | 1.4 | 1.75 | 1.42 |
|  | AAG | 0.31 | 0.25 | 0.09 | 0.47 | 0.06 | 0.14 | 0.37 | 0.42 | 0.41 | 0.6 | 0.25 | 0.58 |
| **Asp** | GAU | 1.37 | 1.45 | 1.54 | 1.40 | 1.70 | 1.66 | 1.66 | 1.4 | 1.39 | 1.23 | 1.72 | 1.56 |
|  | GAC | 0.63 | 0.55 | 0.46 | 0.60 | 0.30 | 0.34 | 0.34 | 0.6 | 0.61 | 0.77 | 0.28 | 0.44 |
| **Glu** | GAA | 1.40 | 1.62 | 1.83 | 1.34 | 1.85 | 1.72 | 1.3 | 1.33 | 1.32 | 1.13 | 1.68 | 0.89 |
|  | GAG | 0.60 | 0.38 | 0.17 | 0.66 | 0.15 | 0.28 | 0.7 | 0.67 | 0.68 | 0.87 | 0.32 | 1.11 |
| **Cys** | UGU | 1.49 | 1.43 | 1.17 | 0.99 | 1.55 | 1.34 | 1.58 | 1.54 | 1.55 | 1.26 | 1.45 | 1.55 |
|  | UGC | 0.51 | 0.57 | 0.83 | 1.01 | 0.45 | 0.66 | 0.42 | 0.46 | 0.45 | 0.74 | 0.55 | 0.45 |
| **Trp** | UGA | 1.47 | 1.70 | 1.87 | 1.67 | 1.77 | 1.73 | 1.42 | 1.49 | 1.5 | 1.13 | 1.74 | 1.32 |
|  | UGG | 0.53 | 0.30 | 0.13 | 0.33 | 0.23 | 0.27 | 0.58 | 0.51 | 0.5 | 0.87 | 0.26 | 0.68 |
| **Arg** | CGU | 0.56 | 1.78 | 1.61 | 1.52 | 0.74 | 0.98 | 1.94 | 2.35 | 2.36 | 1.67 | 1.84 | 1.75 |
|  | CGC | 0.28 | 0.50 | 0.65 | 0.99 | 0.15 | 0.67 | 0.98 | 0.94 | 0.92 | 0.99 | 0.45 | 0.42 |
|  | CGA | 0.54 | 1.16 | 1.20 | 1.70 | 1.41 | 1.22 | 1.01 | 0.96 | 0.95 | 0.87 | 1.5 | 0.99 |
|  | CGG | 0.23 | 0.26 | 0.11 | 0.46 | 0.18 | 0.42 | 0.73 | 0.52 | 0.54 | 1.17 | 0.33 | 0.49 |
| **Ser** | AGU | 0.94 | 1.03 | 0.99 | 0.79 | 0.69 | 1.23 | 1.07 | 0.96 | 0.96 | 1.1 | 1.03 | 1.07 |
|  | AGC | 0.33 | 0.33 | 0.39 | 0.58 | 0.30 | 0.61 | 0.51 | 0.41 | 0.4 | 0.42 | 0.22 | 0.46 |
| **Arg** | AGA | 2.74 | 1.93 | 2.39 | 0.92 | 2.92 | 1.98 | 0.8 | 0.79 | 0.79 | 0.64 | 1.53 | 1.66 |
|  | AGG | 1.66 | 0.36 | 0.04 | 0.42 | 0.59 | 0.74 | 0.54 | 0.44 | 0.43 | 0.66 | 0.36 | 0.7 |
| **Gly** | GGU | 1.73 | 2.13 | 2.03 | 1.57 | 1.10 | 1.49 | 2.05 | 2.05 | 2.05 | 1.84 | 1.92 | 2.11 |
|  | GGC | 0.21 | 0.22 | 0.21 | 0.37 | 0.36 | 0.34 | 0.69 | 0.49 | 0.48 | 0.76 | 0.26 | 0.26 |
|  | GGA | 1.33 | 1.27 | 1.52 | 1.27 | 1.82 | 1.61 | 0.5 | 0.95 | 0.97 | 0.77 | 1.46 | 0.98 |
|  | GGG | 0.72 | 0.38 | 0.24 | 0.79 | 0.71 | 0.56 | 0.76 | 0.5 | 0.5 | 0.63 | 0.36 | 0.65 |

**Table S4** The deviation of actual ENC values from standard curve for protein-coding genes of Spirotrichea representatives

| **Species** | **Deviation** | | |
| --- | --- | --- | --- |
|  | **<=10%** | **10-20%** | **>20%** |
| *Strombidium* cf. *sulcatum* | 0.55 | 0.32 | 0.14 |
| *Oxytricha trifallax* | 0.49 | 0.34 | 0.17 |
| *Laurentiella strenua* | 0.46 | 0.37 | 0.17 |
| *Urostyla grandis* | 0.78 | 0.22 | 0.00 |
| *Pseudokeronopsis carnea* | 0.56 | 0.30 | 0.14 |
| *Pseudokeronopsis flava* | 0.63 | 0.25 | 0.12 |
| *Euplotes vanleeuwenhoeki* | 0.78 | 0.20 | 0.03 |
| *Euplotes minuta* | 0.86 | 0.03 | 0.11 |
| *Euplotes vannus* | 0.78 | 0.18 | 0.05 |
| *Euplotes crassus* | 0.78 | 0.18 | 0.05 |
| *Euplotes raikovi* | 0.75 | 0.18 | 0.07 |
| *Diophrys appendiculata* | 0.80 | 0.12 | 0.07 |
